# Supplementary material for: Tug-of-war between actomyosin-driven antagonistic forces determines the positioning symmetry in cell-sized confinement
Source: Nat Commun. 2020 Jun 15;11:3063. doi: 10.1038/s41467-020-16677-9 (PMC7295813; doi:10.1038/s41467-020-16677-9)
Supplement: Supplementary file 1 — Supplementary Information [file 41467_2020_16677_MOESM1_ESM.pdf]

Supplementary Information for

Tug-of-war between actomyosin-driven antagonistic forces determines the positioning symmetry in cell-sized confinement

Ryota Sakamoto†, Masatoshi Tanabe†, Tetsuya Hiraiwa, Kazuya Suzuki, Shin'ichi Ishiwata, Yusuke T. Maeda, and Makito Miyazaki*

†These authors contributed equally to this work.
*Corresponding author, E-mail: miyazaki.makito.3s@kyoto-u.ac.jp

Supplementary Figures 1-15

Supplementary Notes 1-4

Supplementary References [1]-[5]

Supplementary Figures

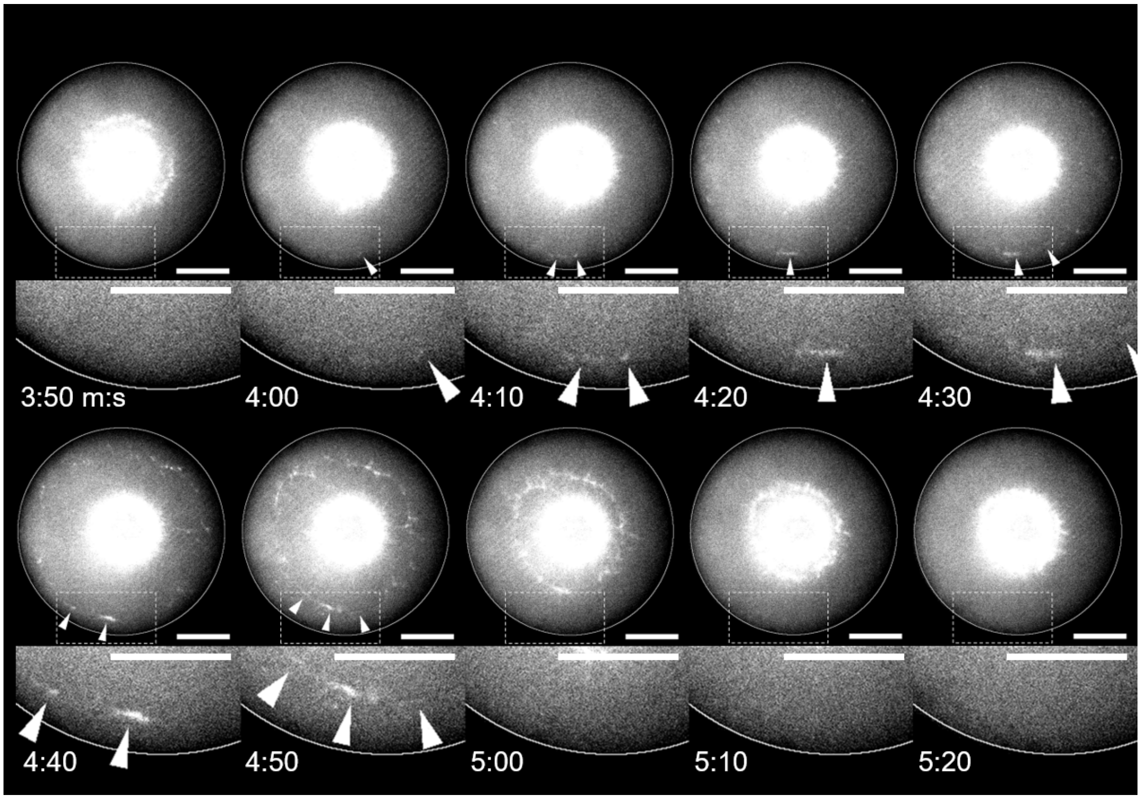

Supplementary Figure 1 | Actomyosin waves are generated near the droplet boundary. Time-lapse confocal images of F-actin near the droplet equator. Arrowheads show newly-formed F-actin networks, indicating that actomyosin waves were generated near the droplet boundary, followed by rapid contraction towards the droplet center. Actin polymerization was initiated at 0 s. Scale bars, 50 μm.

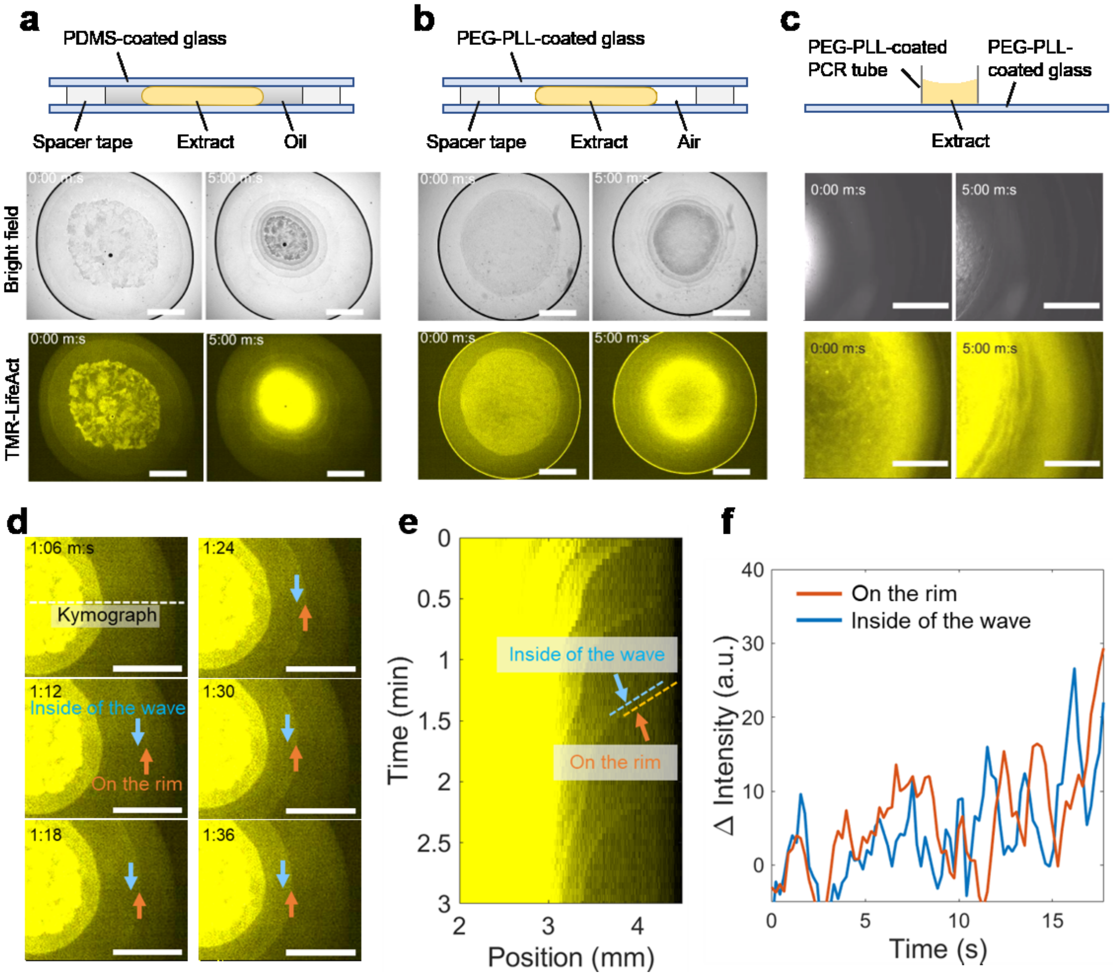

Supplementary Figure 2 | Cluster formation and periodic wave generation in bulk-scale systems. Cluster formation followed by periodic wave generation (**a**) in a mm-sized extract-in-oil droplet, (**b**) in a mm-sized droplet surrounded by air, and (**c**) in a test-tube. (**d**) Time-lapse images and (**e**) the kymograph of **a**. (**f**) Fluorescence intensity along the each broken line in **e**, which also corresponds to the intensity at the arrows with the same color in **d**. Both F-actin densities on the rim and inside of the wave increased during contraction. The thickness of the spacer was 100 μm. Scale bars, 1 mm.

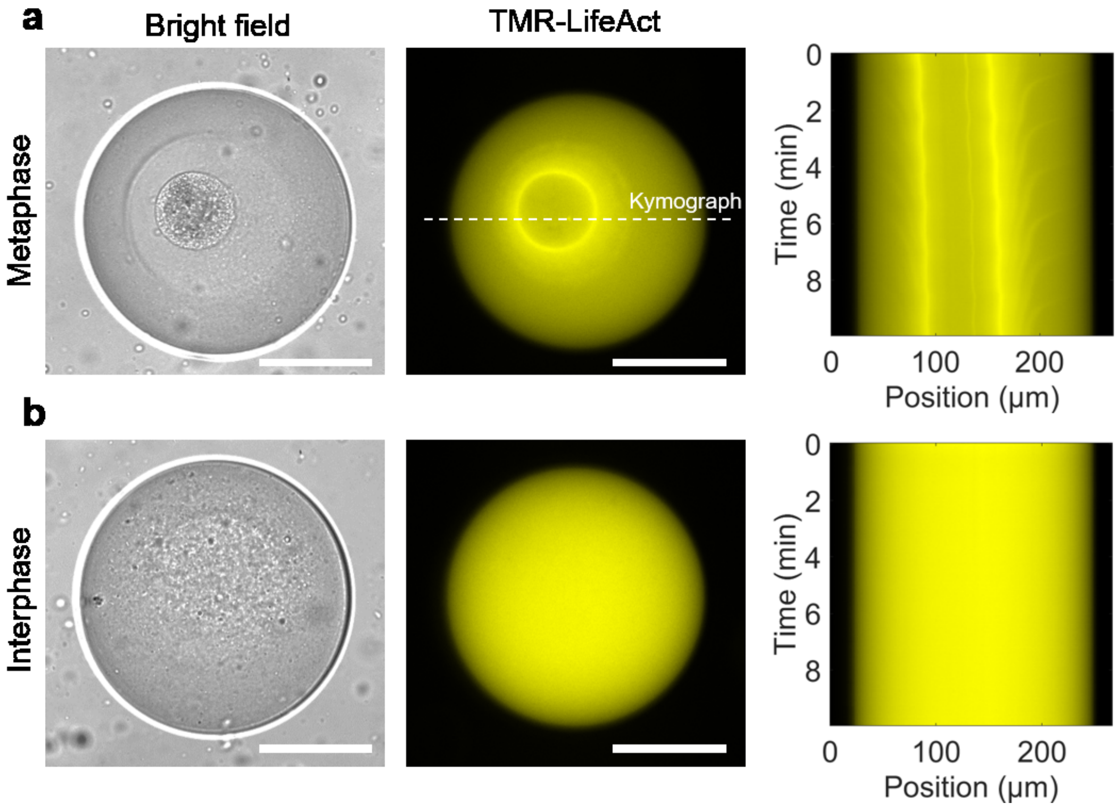

Supplementary Figure 3 | Comparison between metaphase and interphase extracts. (**a**) Metaphase and (**b**) Interphase extracts. The interphase extracts were prepared by treating the metaphase extracts with 0.4 mM $CaCl_2$, followed by 30 min incubation at 20°C. The interphase extracts did not form clear cluster and generate periodic actomyosin waves. Scale bars, 100 μm.

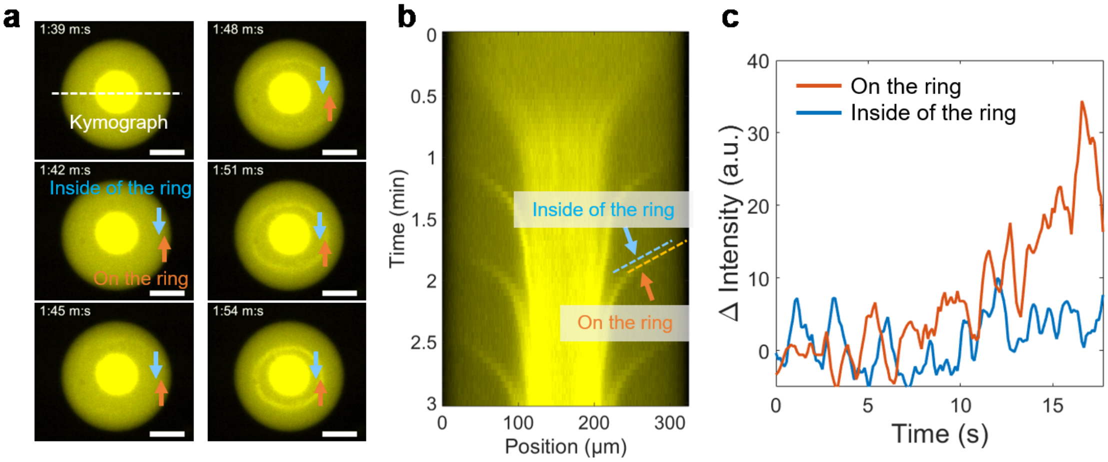

Supplementary Figure 4 | F-actin density of the ring increases as the ring contracts. (**a**) Time-lapse images showing the actomyosin wave propagation. (**b**) The kymograph is extracted from broken line in **a**. (**c**) Fluorescence intensity along the each broken line in **b**, which also corresponds to the intensity at the arrows with the same color in **a**. F-actin density on the ring significantly increases during contraction, whereas F-actin density inside of the ring remains nearly constant. Scale bars, 100 μm.

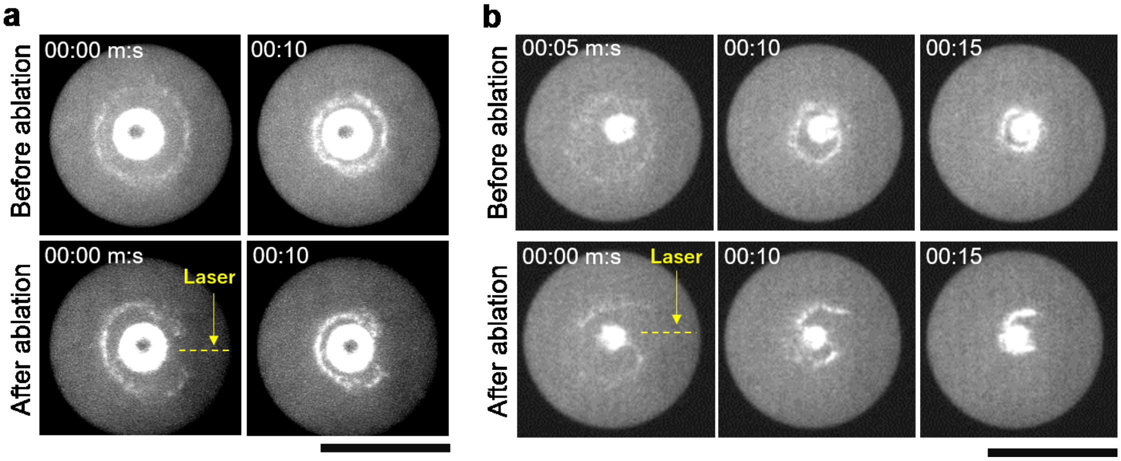

Supplementary Figure 5 | Actomyosin waves opened up just after cutting. A ring-shaped actomyosin wave was cut by a UV pulsed laser during contraction to the droplet center. Just after the laser ablation, the ring was opened up. Typical two examples are shown. Supplementary Movies 4 and 5 correspond to (**a**) and (**b**), respectively. Although the contraction was not completely disrupted by the laser ablation, some opened rings did not contract toward the center, but contracted along the arcs. Scale bars, 100 μm.

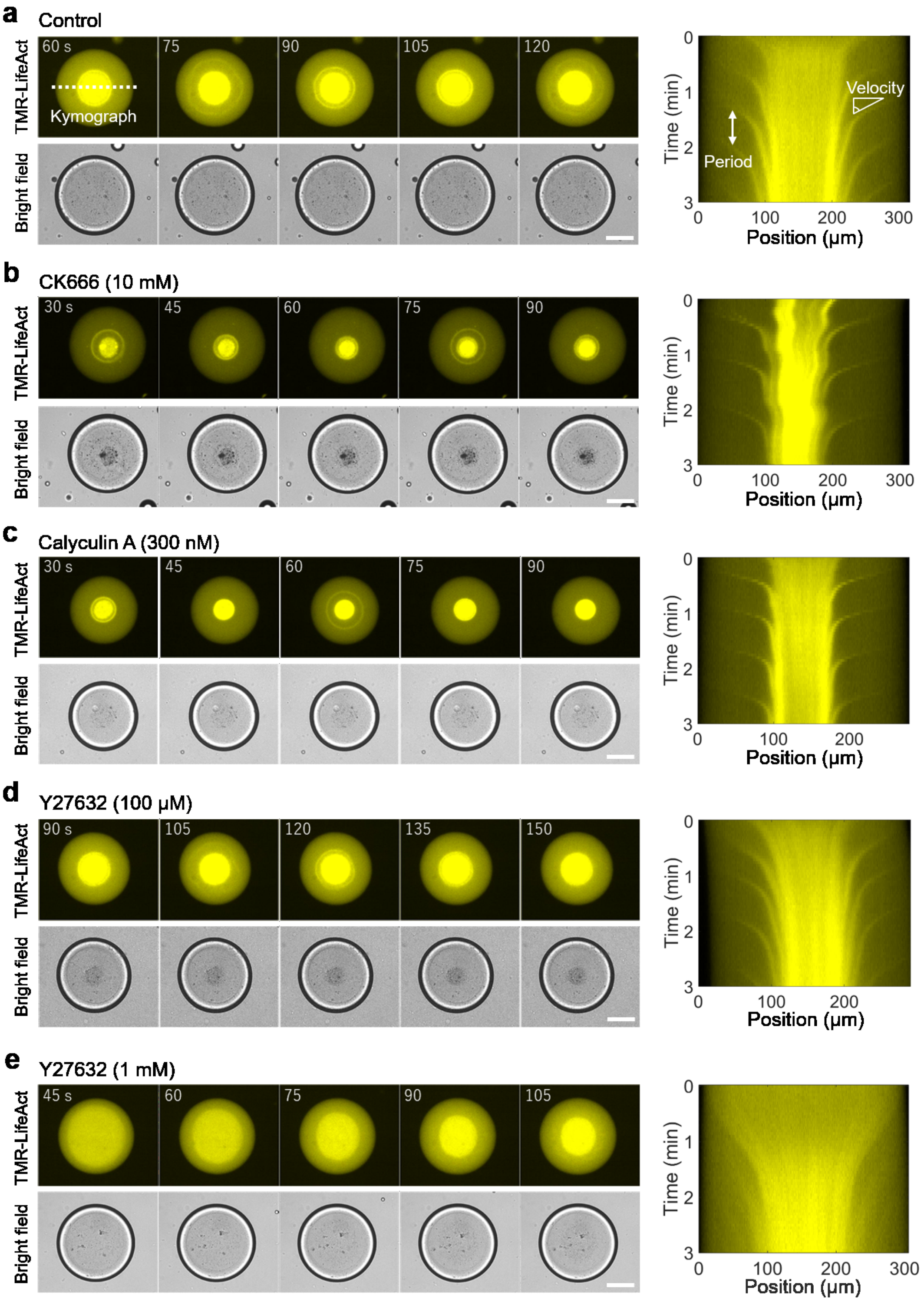
a
Control
TMR-LifeAct
Bright field
60 s
75
90
105
120
Kymograph
Time (min)
Position (μm)
Velocity
Period
b
CK666 (10 mM)
30 s
45
60
75
90
c
Calyculin A (300 nM)
30 s
45
60
75
90
d
Y27632 (100 μM)
90 s
105
120
135
150
e
Y27632 (1 mM)
45 s
60
75
90
105

Supplementary Figure 6 | Partial inhibition and activation of actin nucleation and myosin contraction activities. (**left**) Time-lapse images and (**right**) the kymographs of cluster formation and actomyosin waves. The cluster was formed at 0 s. The actomyosin wave shows a biphasic velocity profile; the initial contraction velocity shows almost constant velocity, while the contraction velocity decreases on approaching the cluster. In this figure, a different batch of the extracts was used from the main text. (**a**) A control experiment (Supplementary Movie 6). Droplet diameter, 317 μm. The wave period and the initial contraction velocity (Supplementary Fig. 7) were analyzed from the kymograph. (**b**) In the presence of CK666 (10 mM) (Supplementary Movie 6). CK666 inhibits actin nucleation activity of the Arp2/3 complex. The period of actomyosin wave exceeded that of the control. Droplet diameter, 314 μm. (**c**) In the presence of calyculin A (300 nM) (Supplementary Movie 6). Calyculin A activates myosin motor activity by inhibiting dephosphorylation of myosin regulatory light chain by phosphatases. The contraction velocity was greater than that of the control experiment. Droplet diameter, 279 μm. (**d**) In the presence of Y27632 (100 μM) (Supplementary Movie 6). Y27632 inhibits myosin motor activity by inhibiting phosphorylation of myosin regulatory light chain by ROCK. The contraction velocity decreased compared to the control experiment. Droplet diameter, 292 μm. (**e**) In the presence of Y27632 (1 mM) (Supplementary Movie 6). The initial cluster formation was significantly decelerated, suggesting that the cluster formation is regulated by myosin. Droplet diameter, 305 μm. Scale bars, 100 μm.

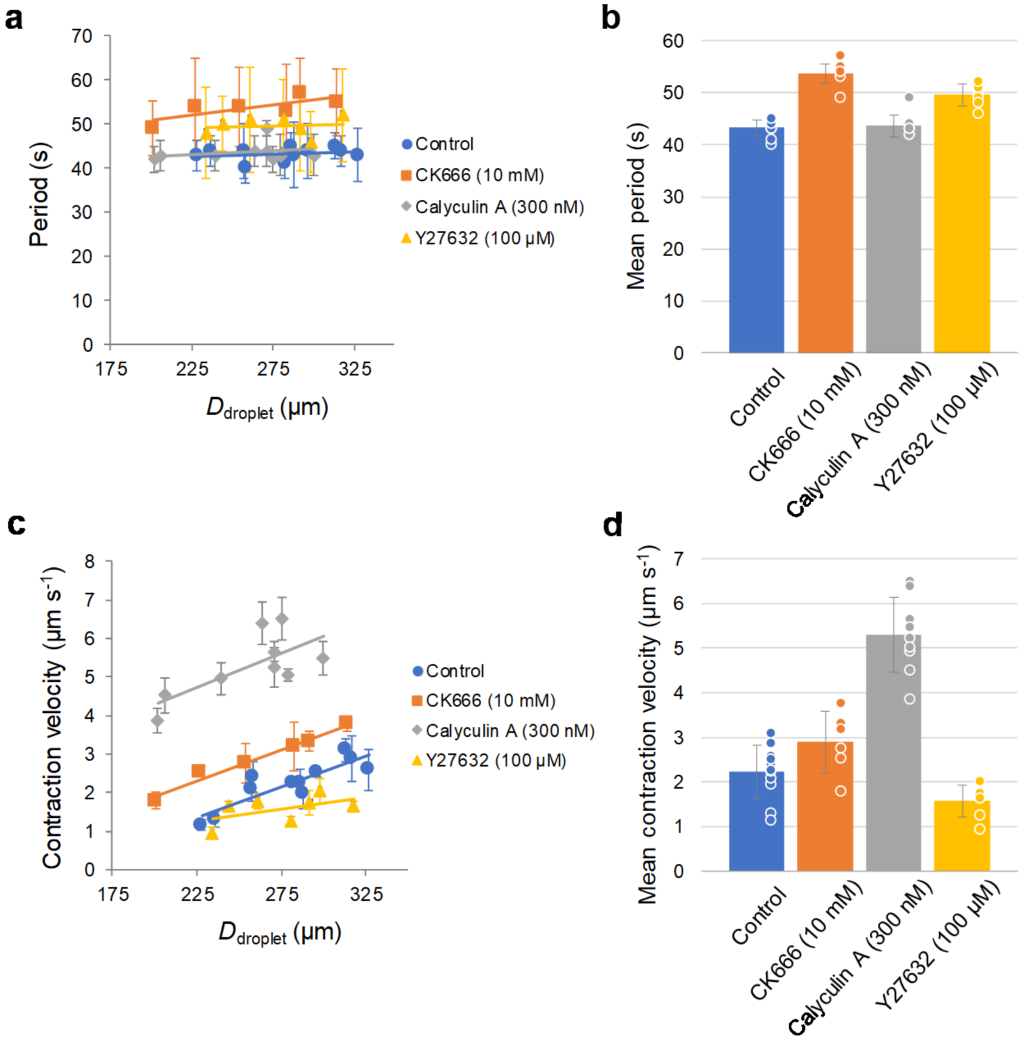

Supplementary Figure 7 | Period and velocity of actomyosin waves in the presence of actomyosin inhibitor/activator (**a**,**b**) Effects of molecular perturbation on the actomyosin wave period. (**a**) The wave periods of each droplet and (**b**) mean values. Inhibition of actin nucleator Arp2/3 by CK666 (10 mM) extended the wave period, indicating that Arp2/3 polymerizes actin filaments at the droplet boundary. Inhibition of myosin by Y27632 (100 μM) slightly extended the period, suggesting that myosin binding on polymerized actin filaments became less efficient owing to the decreased effective myosin concentration. (**a**) The wave period T was

fitted by $T = 1.2 \times 10^{-2} D_{\mathrm{droplet}} + 40$ (Control, n = 11), $T = 4.7 \times 10^{-2} D_{\mathrm{droplet}} + 41$ (CK666 (10 mM), n = 6), $T = 1.6 \times 10^{-2} D_{\mathrm{droplet}} + 40$ (Calyculin A (300 nM), n = 9), $T = 0.94 \times 10^{-2} D_{\mathrm{droplet}} + 47$ (Y27632 (100 μM), n = 7). Error bars represent standard deviations (SDs) from the mean periods averaged over three successive wave periods. (**b**) The mean wave periods averaged over the droplets shown in **a**. Error bars represent SDs. The corresponding data points are plotted as the circles. (**c,d**) Effects of molecular perturbation on the contraction velocity of actomyosin waves. (**c**) The initial contraction velocities of each droplet and (**d**) the mean values averaged over the droplets shown in **c**. Myosin activation by calyculin A (300 nM) drastically increased the velocity, while inactivation by Y27632 (100 μM) decreased the velocity, suggesting that myosin is involved in wave propagation. (**c**) The initial contraction velocity v was fitted by $v = 1.6 \times 10^{-2} D_{\mathrm{droplet}} - 2.2$ (Control, n = 11), $v = 1.6 \times 10^{-2} D_{\mathrm{droplet}} - 1.3$ (CK666 (10 mM), n = 6), $v = 1.8 \times 10^{-2} D_{\mathrm{droplet}} + 0.75$ (Calyculin A (300 nM), n = 9), $v = 0.63 \times 10^{-2} D_{\mathrm{droplet}} - 0.16$ (Y27632 (100 μM), n = 7). Error bars represent SDs from the mean velocity averaged over three successive waves. (**d**) The mean contraction velocity averaged over the droplets shown in **c**. Error bars represent SDs. The corresponding data points are plotted as the circles.

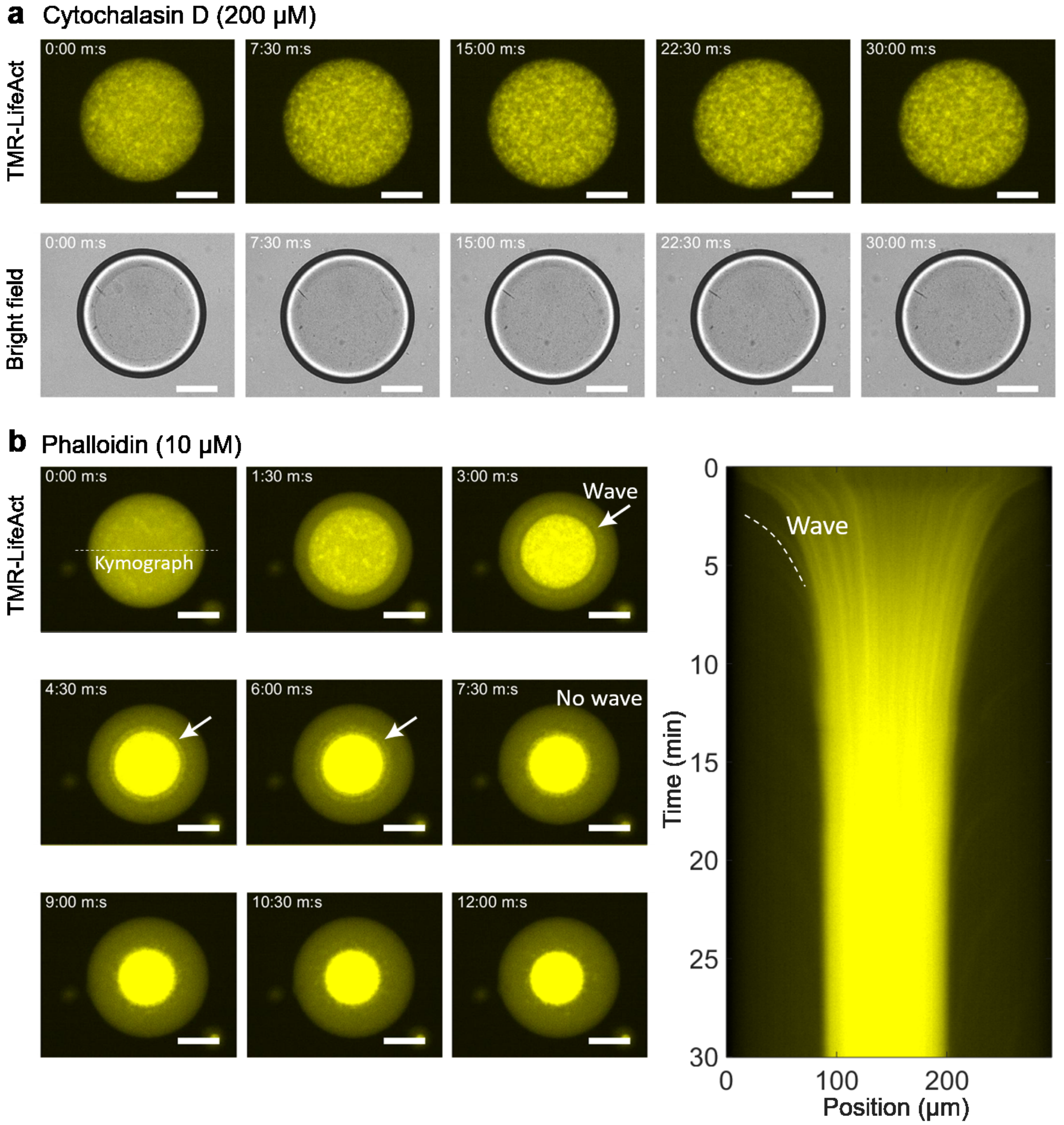

Supplementary Figure 8 | Continuous actin filament turnover is essential for periodic actomyosin wave generation. (**a**) Addition of cytochalasin D (200 μM), an actin polymerization inhibitor, eliminated cluster formation and actomyosin wave generation (Supplementary Movie 7). (**b**) Addition of phalloidin (10 μM), an actin depolymerization inhibitor, decreased the initial actin network contraction

velocity and eliminated actomyosin wave generation after two wave propagations (see arrows and Supplementary Movie 8). Actin polymerization was initiated at 0 s. Scale bars, 100 μm.

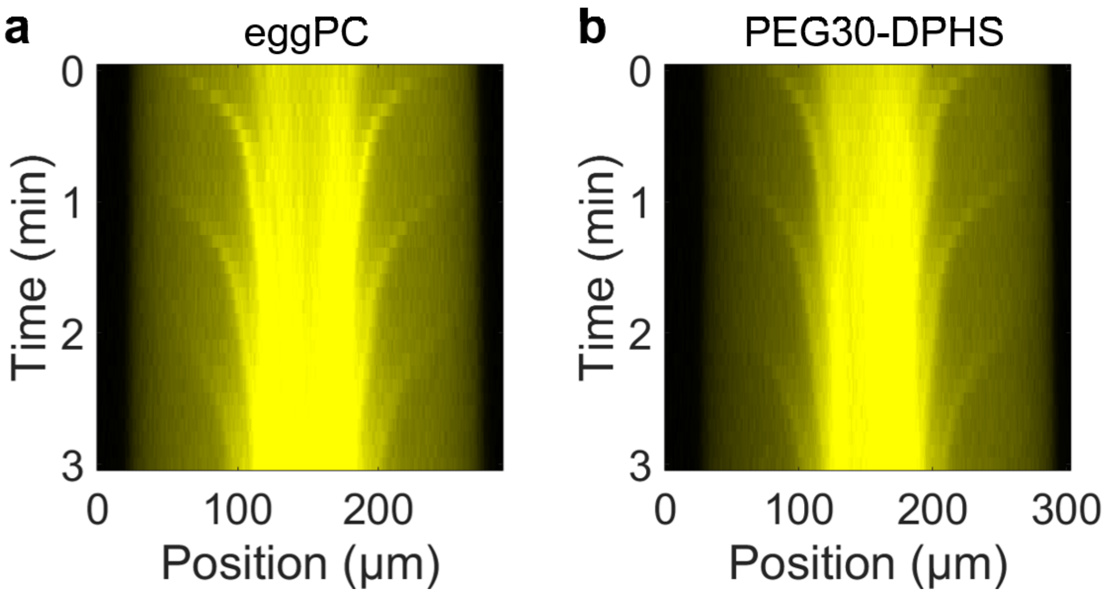

Supplementary Figure 9 | Effect of surface passivation on wave generation. Kymograph of the time-lapse TMR-LifeAct images of the droplet surrounded by (**a**) eggPC or (**b**) PEG30-DPHS. The periodic waves were generated in both cases.

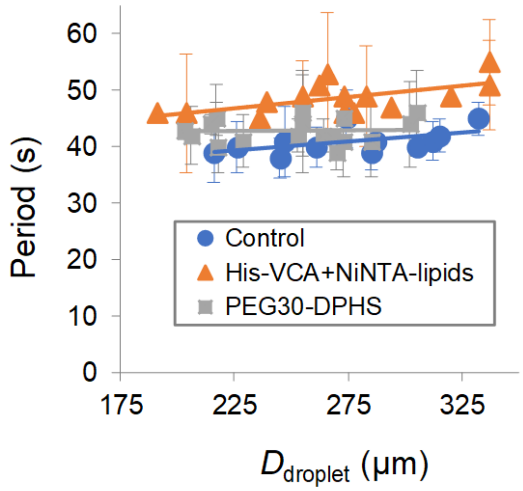

Supplementary Figure 10 | Wave period under different droplet surfaces. Comparison of the wave periods among the droplets having different surface properties. The wave period T was fitted by $T = 3.2 \times 10^{-2} D_{\mathrm{droplet}} + 32$ (Control, n = 12), $T = 4.1 \times 10^{-2} D_{\mathrm{droplet}} + 38$ (His-VCA+NiNTA-lipids, n = 15), $T = 3.8 \times 10^{-3} D_{\mathrm{droplet}} + 42$ (PEG30-DPHS, n = 18). Error bars represent standard deviations (SDs) from the mean periods averaged over three successive wave periods. Note that the different batch of the extracts from the main text was used.

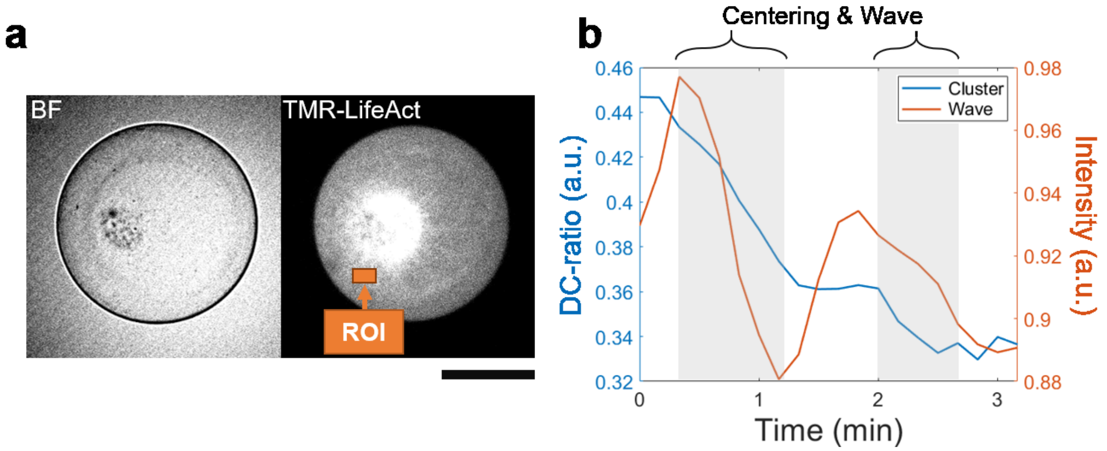

Supplementary Figure 11 | Relationship between wave propagation and cluster motion. (**a**) Images of the droplet used for analysis and (**b**) correlation between the wave and the cluster motion. When the wave approached the cluster, the cluster moved toward the center. The cluster motion was analyzed from the bright field image. The wave intensity was measured from the ROI in TMR-LifeAct image. Scale bar, 100 μm.

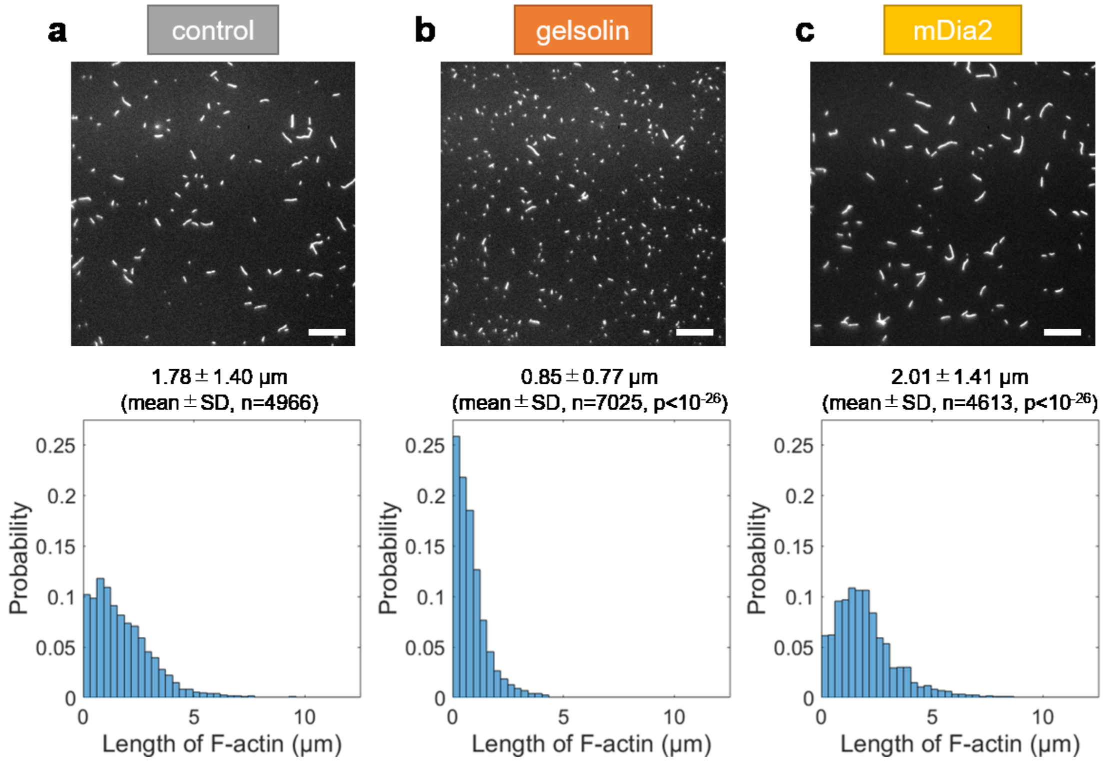

Supplementary Figure 12 | Measurements of the length distribution of F-actin. The length distributions of F-actin is measured by fluorescence microscopy. F-actin is fixed and visualized by rhodamine-phalloidin. The length distribution of gelsolin and mDia2 are significantly different from the control experiment (p-values are displayed; Kolmogorov-Smirnov test). (**a**) Control experiment. Mean F-actin length: 1.78 ± 1.40 μm (sample size, $n = 4966$). (**b**) Addition of 300 nM gelsolin. Mean F-actin length: 0.85 ± 0.77 μm ($n = 7025, p < 10^{-26}$). (**c**) Addition of 30 nM mDia2. Mean F-actin length: 2.01 ± 1.41 μm ($n = 4613, p < 10^{-26}$). Scale bars, 10 μm.

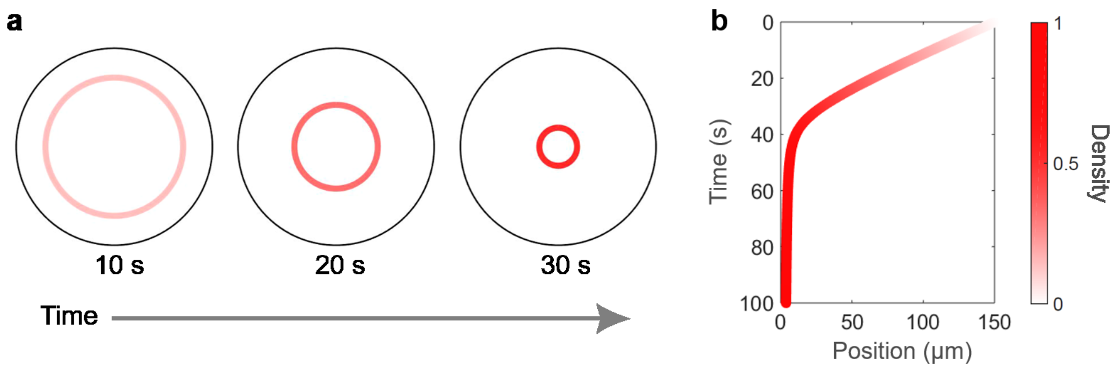

Supplementary Figure 13 | Numerical simulation of the actomyosin wave propagation (**a**) Time evolution of the actomyosin wave and (**b**) the kymograph. The wave was simulated in accordance with the active gel model with the following parameters: $v_0 = 5.0$ (μm s^{-1}) and $r_0 = 30$ (μm) (Supplementary Note 1).

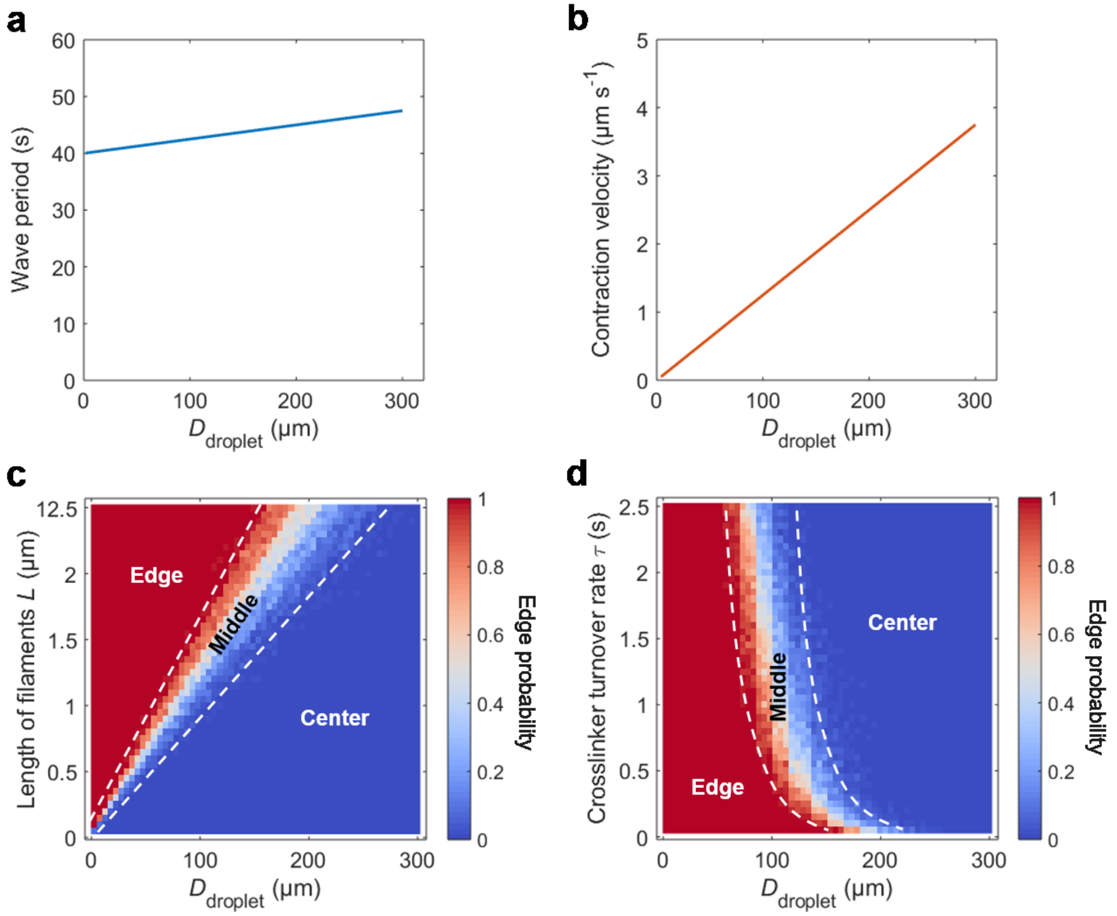

Supplementary Figure 14 | Numerical simulation of the tug-of-war model. (**a,b**) Wave period and contraction velocity used for numerical simulations of the cluster positioning. The size-dependent velocity of the actomyosin wave and the size-dependent wave period were set as $v = 1.25 \times 10^{-2} D_{\text{droplet}}$ and $T = 2.5 \times 10^{-2} D_{\text{droplet}} + 40$, respectively. It was assumed that the contraction velocity of the actomyosin bridge was equal to the velocity of the actomyosin wave. (**c,d**) The phase diagrams of cluster positioning. (**c**) The turnover rate of the crosslinkers was fixed as $\tau = 0.5$ s, and filament length L was varied. (**d**) The filament length was fixed as $L = 8$ μm, and the turnover rate of the crosslinkers τ was varied (Supplementary Note 4).

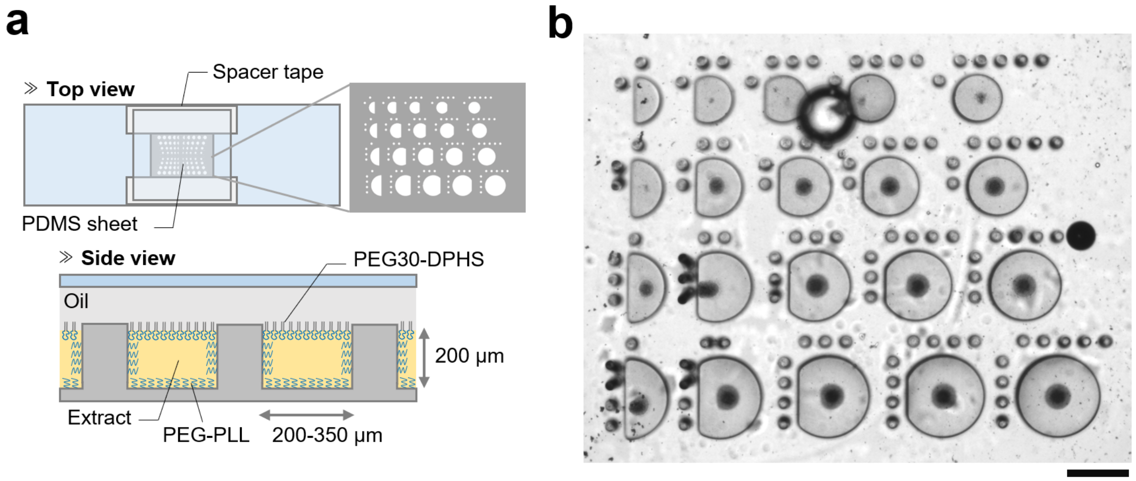

Supplementary Figure 15 | Cluster positioning in non-circular geometries. (**a**) Schematic illustration of the experimental setup. The extracts were confined in patterned PDMS chambers and sealed by the oil containing PEG30-DPHS. The surface of PDMS chambers had been coated by PEG-PLL, Therefore, both the extract-PDMS interface and the extract-oil interface were passivated with PEG. (**b**) A bright-field image of the extracts confined in asymmetric chambers. Most of the clusters positioned near the center even if the chamber was non-circular shape. Scale bar, 300 μm.

Supplementary Notes

Supplementary Note 1: Active gel model for the contraction velocity of an actomyosin wave

In this section, we describe the dynamics of actomyosin wave propagation by an active gel theory [1]. The basis of the idea here is as follows: (i) gelation-and-contraction mechanism is assumed for the initiation of the wave [2], and (ii) after the actomyosin network shrinks and its density becomes higher, the network relaxation time becomes larger exponentially, which leads to the huge drop of contraction speed. While this idea can be applicable to both cases that the contractile actomyosin network is homogeneously spread within the droplet (i.e. bulk gelation contraction [2]) and that it shapes the ring near the droplet boundary, we quantify the latter case by reflecting our experimental observation.

Given that an actomyosin ring (a hollow cylinder) of constant radial thickness d is confined in a cylindrical chamber of height h. We derive the ring radius r-dependence of contraction velocity v as follows. The volume V of the thin hollow cylinder of radius r (assuming $r \gg d$) is written as $V \sim 2\pi r d h$; hence, the actomyosin density ρ is given by

$$\rho = \frac{M_0}{2\pi d h}\frac{1}{r} \propto \frac{1}{r} \tag{1}$$

with the constant mass M_0 of the actomyosin. The density of the actomyosin ρ increases with time t because the ring radius r decreases by myosin-induced contractile stress, and it is assumed that actin filaments are not depolymerized during contraction.

Simultaneously with ring shrinkage, motor-induced contractile stress σ increases with an increase in myosin density. The sign of σ is defined such that it is positive for contractile active stress. We here specifically assume that the strength of active stress is proportional to the density of myosin. Furthermore, we assume in this section (Supp. Note 2), that both actin and myosin densities are proportional to the total actomyosin density ρ. Thus, we have $\sigma = E_\rho \rho$, and hence

$$\sigma = \frac{E_r}{r} \tag{2}$$

with proportionality factors E_ρ and $E_r = E_\rho M_0/(2\pi d h)$.

In addition, actin-crosslinking proteins are contained in the cytosol. Given that new crosslinkers bind to the filament with the average rate $k_{\rm on}$ and each crosslink is disconnected with the average lifetime $\tau_{\rm c}$, the characteristic relaxation time of the

actomyosin network is given by

$$\tau = \frac{1}{k_{\rm on}}(e^{k_{\rm on}\tau_{\rm c}} - 1). \tag{3}$$

Supplementary Equation (3) is derived as follows (see ref. [3] for the detailed derivation): Given that an actin filament has n-crosslinked points, we will derive the average time T_n that all crosslinkers are stochastically detached from the actin filament (i.e., the mean first passage time from the n-crosslinked state to 0-crosslinked state). The recurrence formula is written as

$$T_n = \frac{k_{\rm on}}{k_{\rm on} + n/\tau_{\rm c}} T_{n+1} + \frac{n/\tau_{\rm c}}{k_{\rm on} + n/\tau_{\rm c}} T_{n-1} + \frac{1}{k_{\rm on} + n/\tau_{\rm c}}. \tag{4}$$

Here, we take the following boundary conditions: (i) $T_0 = 0$ and (ii) $T_n - T_{n-1} \to 0$ for $n \to \infty$. Thus, Supplementary Equation (4) is rewritten as

$$k_{\rm on}(T_n - T_{n+1}) + \frac{n}{\tau_{\rm c}}(T_n - T_{n-1}) = 1. \tag{5}$$

From Supplementary Equation (5), after a few manipulations [3], we obtain

$$T_n = \sum_{k=0}^{n-1} \frac{k!}{(k_{\rm on}\tau_{\rm c})^k} \sum_{m=k+1}^{\infty} \frac{(k_{\rm on}\tau_{\rm c})^m}{k_{\rm on} m!}. \tag{6}$$

Substituting $n = 1$ into Supplementary Equation (6),

$$T_1 = \frac{1}{k_{\rm on}}(e^{k_{\rm on}\tau_{\rm c}} - 1). \tag{7}$$

Since the characteristic relaxation time of the actomyosin network τ is defined by the time during which an actin filament having single crosslinked point is detached from another filament, we obtain Supplementary Equation (3).

Assuming that the binding rate of an actin filament to another filament $k_{\rm on}$ is proportional to the density of actin, Supplementary Equation (3) can be rewritten as

$$\tau = \frac{\tau_{\rm c}}{B\rho}(e^{B\rho} - 1) \tag{8}$$

with a proportionality coefficient B of $k_{\rm on}\tau_{\rm c}$ for ρ. Note that the coefficient of viscosity μ is proportional to the relaxation time τ, therefore

$$\mu = G\frac{1}{B\rho}(e^{B\rho} - 1), \tag{9}$$

where G is a proportionality coefficient having dimensions of viscosity.

Given that circumference of the cylindrical actomyosin ring is $C = 2\pi r$, the force balance $\sigma = -\mu\dot{\epsilon}$ with the strain rate $\dot{\epsilon} \equiv (1/C)(dC/dt)$ is given by

$$\sigma = -\mu \frac{1}{r}\frac{dr}{dt} \ . \tag{10}$$

Substituting Supplementary Equations (1), (2) and (9) into Supplementary Equation (10), the ring radius r-dependence of contraction velocity $v \equiv -dr/dt$ is given by

$$v = v_0 \left(\frac{r_0}{r}\right) \left[\exp\left(\frac{r_0}{r}\right) - 1\right]^{-1} , \tag{11}$$

where $v_0 = E_r/G$ and $r_0 = BM_0/(2\pi dh)$. In the main text, we set v_0 and r_0 as the fitting parameters. Note that the velocity of a small actomyosin ring decays with time in accordance with $v \sim \exp(-r_0\kappa)$, where $\kappa = 1/r$ is the ring curvature. It is difficult for condensed actomyosin networks to reorganize their configuration, thus increasing the relaxation time and viscosity at the actomyosin ring, causing exponential decay of the contraction velocity.

As long as the pure gelation-contraction mechanism is assumed, the initial contraction velocity $v_{c0} \equiv -dr/dt|_{r=R}$ is proportional to the droplet radius R,

$$v_{c0} \propto R \ , \tag{12}$$

reflecting that the contractile strain rate at the initial time $\dot{\epsilon}_0 \equiv (1/C(t=0))(dC/dt(t=0))$ with $C(t=0) = 2\pi R$ and $C(t) = 2\pi r(t)$ is constant (Supplementary Equation (12) is applicable even when the active gel is not the cylinder associated with the droplet boundary but homogeneously spread around the droplet bulk). However, in our experimental setup, an interaction between the droplet boundary and active gel is not negligible. This may also affect quantitatively the gelation-and-contraction mechanism. We will further discuss this correction in the size-dependency of the initial contraction velocity at the latter half of Supplementary Note 2.

Supplementary Note 2: Weak correction for the wave contraction period and velocity due to interaction between the droplet boundary and active gel

In this section, we derive the contribution of interaction between the droplet boundary and active gel to the wave contraction period and velocity. We find the weak

correction in the wave contraction period and velocity compared to the pure gelation-and-contraction case, as seen in the experimental results (Figs. 1 i and j in the main text).

Wave contraction period. We firstly consider active gel dynamics in a cylindrical chamber of height h. Given that actin network of thickness d is polymerized on the periphery of the cylindrical chamber. Note that the filamentous actin network can be well-connected after time t_0, at which actin filaments are long enough to transmit forces over a long distance. Running over the time t_0, polymerized long actin filaments gets contact points with each other. Subsequently, myosin and other crosslinkers associate on the actin filaments with time, forming inter-connected actomyosin networks, thereby increasing the contractile stress in proportion to the density of myosin bound on the network. Together, time dependence of the contractile stress σ is written as

$$\sigma = \begin{cases} 0 & (t < t_0) \quad (13a) \\ \alpha(t - t_0) & (t > t_0), \quad (13b) \end{cases}$$

where α is the rate of association of myosin onto the network.

We then consider the contraction period T of the actomyosin network. Given that the radius of the cylindrical chamber is R, stress applied on the chamber f_{wall} is calculated using the following force balance equation:

$$f_{\text{wall}} R A(R) = \sigma V(R), \tag{14}$$

where $A(R) = 2\pi Rh$ is the lateral area of the cylindrical actomyosin, and $V(R) = 2\pi Rhd$ is the volume of the actomyosin (see Supplementary Reference [4] for details). Thus, stress applied on the chamber is written as

$$f_{\text{wall}} = \frac{\sigma d}{R} = \frac{\alpha d}{R}(t - t_0), \tag{15}$$

and the network shrinks when stress exceeds a threshold f_{th} to disrupt anchoring between the chamber and the network, followed by polymerization on the periphery of the chamber. Therefore, the contraction period of the actomyosin network is given by

$$T = t_0 + \frac{f_{\text{th}}}{\alpha d} R. \tag{16}$$

Wave contraction speed. We here derive the initial contraction velocity of the actomyosin wave upon initiation of its shrinkage. Contractile stress at the beginning

of shrinkage is given by Supplementary Equation (15) as $\sigma_{c0} = f_{\mathrm{th}} R/d$. We can thus calculate the initial contraction velocity $v_{c0} \equiv -dr/dt|_{r=R}$ by using Supplementary Equations (9) and (10) and obtain, instead of Supplementary Equation (12),

$$v_{c0} = \frac{\sigma_{c0}}{\mu} R = \frac{f_{\mathrm{th}} B \rho_{c0}}{Gd} R^2 [e^{B\rho_{c0}} - 1]^{-1}, \tag{17}$$

where ρ_{c0} is the density at the beginning of shrinkage and can be expressed as $\rho_{c0} = (R/d)(f_{\mathrm{th}}/E_\rho)$ from Supplementary Equation (15) with $\sigma = E_\rho \rho$. (Remember that this proportionality of σ upon ρ has been introduced previously in this section.) Therefore, we obtain the following chamber radius R-dependence of the initial contraction velocity v_{c0}:

$$v_{c0} = \frac{H f_{\mathrm{th}}}{Gd} \frac{R^3}{\exp(HR) - 1}, \tag{18}$$

where we introduced the coefficient $H = (B f_{\mathrm{th}})/(E_\rho d)$, yielding $B\rho_{c0} = HR$. Thus, the interaction between droplet boundary and active gel can modulate the initial contraction velocity from the simple proportionality $v_{c0} \propto R$.

Supplementary Note 3: Tug-of-war model of cluster positioning

In this section, we derive the probability that a cluster is at the edge, based on the percolation theory [5]. For simplicity, we assume that (i) the cluster is transported to the droplet center by actomyosin waves generated within a time period of T, and that (ii) the cluster is started to be contracted to the edge if actomyosin in bulk space forms a network bridge between the cluster and the droplet boundary (wall of the cylindrical chamber), and movement is stopped when the cluster collides with the subsequent actomyosin wave. We suppose the cluster is finally in the edge in the case of high percolation probability during the period T; otherwise, it is in the center. We then calculate the probability that the actin filaments of length L aligned between the cluster and droplet boundary are connected with crosslinkers during period T. Given that the distance between the cluster and droplet boundary is R (For a droplet of diameter D, $R \simeq D/2$), the actin filaments percolates and connects the cluster and droplet boundary when all the $N \equiv R/L$ actin-crosslinking sites are occupied by crosslinkers.

For analytical expression of the model, we assume here that the cluster immediately moves to the edge if percolation occurs during the period T; otherwise, it is positioned at the center (two-state model). Suppose that each crosslinker binds and

unbinds at a crosslinking site with binding and unbinding rates $k_{\rm on}$ and $k_{\rm off}$, respectively. The characteristic time within which all crosslinkers on one actin filament undergo turnover is given by $\tau = 1/(k_{\rm off} + C_0 k_{\rm on})$, where C_0 denotes the crosslinker concentration in solution. In the simplest case, assuming the equilibrium between the association and dissociation of crosslinkers, each crosslinking sites take "on" or "off" states independently for every time interval τ with a probability of 1/2. Then the probability that all N sites are occupied is given by $(1/2)^N$ at a specified timepoint. Thus, we obtain the probability that all N crosslinking sites are occupied by crosslinkers at least one time during the time period T as

$$p = 1 - [1 - (1/2)^N]^{T/\tau}. \tag{19}$$

This is the probability that the cluster is entrapped in the edge region. Here we may use $N = R/L$ with the typical maximum length L of the actin filaments because of the following facts: (i) Firstly, short filaments can hardly line up across the center to the periphery of the droplet over the radius R by chance. Given that there are one filament of length $L = R$ with the probability distribution of 0.01, and five filaments of length $L = R/5$ with the probability distribution of 0.1. The probability that the five shorter filaments of $R/5$ are aligned in a line from center to the boundary over the distance of R is $(0.1)^5$, and this probability is significantly smaller than the probability that the long filament of R exists in the same direction, which is equal to 0.01. (ii) In addition, the typical percolation probability via crosslinker binding decreases exponentially for decreased actin length L. Given that the filament of length $L = R$ and $L = R/5$, we can estimate the percolation probability as $(1/2)^{R/L} = (1/2)^1$ and $(1/2)^5$, respectively. Altogether, for the percolation probability, only the maximally long filaments may play the dominant role.

Substituting $N = R/L = D/(2L)$ into Supplementary Equation (19) reads droplet diameter D-dependence of the percolation probability:

$$p(D) = 1 - [1 - (1/2)^{D/(2L)}]^{T(D)/\tau} \tag{20}$$

Note that Supplementary Equation (20) can be written as $p \simeq 1 - \exp[-(T(D)/\tau)(1/2)^{D/(2L)}]$ for sufficiently large N. Thus, the percolation probability decreases from 1 to 0 with an increase in droplet size, reproducing the experimentally observed size-dependent cluster positioning.

Here we expect that only very long filaments, which is a small fraction in the distribution, predominantly contribute to the percolation. In the proposed model, the probability that a bridge is formed by the length L of F-actin in a droplet with radius R is written as $P = (1/2)^{R/L}$. If we compare the probability with different

actin length, $L = 10$ μm and $L = 5$ μm, in a droplet with radius $R = 50$ μm, we can estimate $P = (1/2)^5$ and $P = (1/2)^{10}$ respectively. Thus, only 2 times change in F-actin length decreases the bridge formation probability $\sim$30 times. To compare with the experimentally obtained actin length, we took only top 5% of the length distribution of F-actin, because the probability of the bridge formation mediated by short F-actin is expected to be much smaller than the longer filaments. Indeed, the F-actin length $L = 5.7$ μm, calculated from averaging top 5% of the length distribution of F-actin, is close to the value $L = 6.7$ μm obtained from the model fitting to the cluster positioning.

Supplementary Note 4: Stochastic simulation of cluster positioning

In this section, we describe the scheme of stochastic simulation of cluster positioning. The basic setup and the meaning of parameters are the same as those in Supplementary Note 3, the only difference being that here we consider cluster movement in space, which was not described above to obtain an analytical solution Supplementary Equation (20). For simplicity, a cluster is assumed to be contracted at the edge region when the actomyosin network bridge is formed between the cluster and the droplet boundary. We implement this in our numerical simulation as follows: In every time steps of τ, each crosslinking site takes an "on" or "off" state with equal probabilities, 1/2. On repeating this trial, the formed actomyosin bridge starts contracting the cluster toward the edge when all N sites are occupied until the cluster meets the actomyosin wave. The contraction rate is assumed to be equal to the velocity of the actomyosin waves, v. Simultaneously, actomyosin waves are generated on the boundary every time period of T, and they propagate toward the droplet center with a velocity of v. Network bridge contraction is stopped and the network connection is disrupted if the migrating cluster collides with the wave, subsequently the cluster is transported toward droplet center by the wave. If the percolation occurs during transportation by the wave, the formed actomyosin bridge again contracts the cluster toward the edge.

We performed simulations for each 20-μm diameter of droplet from $D = 20$ to 300 μm. The length of actin filaments was set as $L = 4, 8$ and 12 μm, and the turnover rate was set as $C_0 = 0.1, 1$ and 10 μM (with turnover rate of crosslinkers: $\tau = (1/(k_{\rm off} + C_0 k_{\rm on})$, where $k_{\rm off} = 0.66$ s^{-1} and $k_{\rm on} = 1.2 \times 10^6$ M^{-1} s^{-1}). The velocity of the actomyosin wave and network bridge contraction was set as $v = 1.25 \times 10^{-2} D$ μm s^{-1}, and the size-dependent wave period was set as $T = 2.5 \times 10^{-2} D + 40$ s to have the same order and dependency on droplet size as the control experiment

(Supplementary Fig. 14). We repeated the simulation run 100 times, using different random seeds for every droplet diameter, in which each simulation corresponded to one sample set of the cluster and droplet. The simulation run for each droplet was finished after time $T_0 = 1800$ s, corresponding to the typical time to reach the steady-state in the experiment. Thereafter, we calculated the time-averaged DC-ratio as $\langle \text{DC-ratio} \rangle_t = (1/T_0) \int_0^{T_0} [d(t)/R] dt$, where $d(t)$ is the distance between the cluster and the droplet center, and R is the droplet radius $R = D/2$. We used time-averaged DC-ratio to evaluate the statistical property of the percolation process. The position is classified in accordance with the value of $\langle \text{DC-ratio} \rangle_t$ as the center ($\langle \text{DC-ratio} \rangle_t < 0.2$), edge ($\langle \text{DC-ratio} \rangle_t \leq 0.8$), and middle ($0.2 < \langle \text{DC-ratio} \rangle_t < 0.8$). We considered 0.8, not 0.5, as the DC-ratio threshold of the edge region in the numerical simulation (Fig. 7). This value was justified by the following calculation: A cluster was assumed as a point particle in numerical simulation, whereas the clusters in experiments had finite size of $D_{\text{cluster}} = 0.26 D_{\text{droplet}}$ (Fig. 1h). We took into account this steric effect by assuming the excluded volume layer from the boundary. We thus included the excluded size $D_{\text{cluster}}/D_{\text{droplet}} \sim 0.3$ to the threshold used in experiments ($0.8 - 0.3 = 0.5$). In contrast, since a cluster in numerical simulation was assumed as a point particle, we used 0.8 instead of 0.5 as the DC-ratio. The DC-ratio for each droplet is summarized as a histogram, and the fraction of clusters in the edge (edge-probability) is displayed with the analytical solution [Supplementary Equation (19)] using the same parameter sets as the numerical simulation (Fig. 7).

Supplementary References

[1] Prost, J., Jülicher, F. & Joanny, J. F. Active gel physics. *Nat. Phys.* **11,** 111-117 (2015).

[2] Field, C. M. *et al.* Actin behavior in bulk cytoplasm is cell cycle regulated in early vertebrate embryos. *J. Cell Sci.* **124,** 2086-2095 (2011).

[3] Hiraiwa, T. & Salbreux, G. Role of turnover in active stress generation in a filament network. *Phys. Rev. Lett.* **116,** 188101 (2016).

[4] Carlsson, A. E. Contractile stress generation by actomyosin gels. *Phys. Rev. E* **74,** 051912 (2006).

[5] Stauffer, D., & Aharony, A. Introduction to Percolation Theory (Taylor & Francis, 1991).
