# Supplementary material for: Tug-of-war between actomyosin-driven antagonistic forces determines the positioning symmetry in cell-sized confinement
Source: Nat Commun. 2020 Jun 15;11:3063. doi: 10.1038/s41467-020-16677-9 (PMC7295813; doi:10.1038/s41467-020-16677-9)
Supplement: Supplementary file 3 — Description of Additional Supplementary Information [file 41467_2020_16677_MOESM3_ESM.pdf]

Description of Additional Supplementary Files

File Name: Supplementary Movie 1
Description: Time-lapse movie of cluster formation and the periodic actomyosin wave in a control experiment. The height of the chamber was 100 µm. Actin polymerization was initiated at 0 s by the elevation of temperature. Scale bars, 100 µm.

File Name: Supplementary Movie 2
Description: Time-lapse movie of the periodic actomyosin wave observed by a confocal microscope, showing persistent wave generation for more than 90 min. The height of the chamber was 60 µm. Scale bar, 50 µm.

File Name: Supplementary Movie 3
Description: Time-lapse movie of the cluster formation and periodic wave generation in mm-scale systems. The height of the chamber was 100 µm. Scale bars, 100 µm.

File Name: Supplementary Movie 4
Description: Time-lapse movie of the actomyosin wave generation before and after the laser cut observed by a confocal microscope. The height of the chamber was 60 µm. Scale bars, 100 µm.

File Name: Supplementary Movie 5
Description: Time-lapse movie of the actomyosin wave generation before and after the laser cut observed by a confocal microscope. The timing of the laser ablation was indicated in the movie. The height of the chamber was 60 µm. Scale bar, 100 µm.

File Name: Supplementary Movie 6
Description: Time-lapse movie of the droplet for control experiments (Supplementary Figure 3a), 10 mM CK666 (Supplementary Figure 3b), 300 nM calyculin A (Supplementary Figure 3c), 100 µM Y27632 (Supplementary Figure 3d), and 1 mM Y27632 (Supplementary Figure 3e). The cluster was assembled at 0 s. The height of the chamber was 100 µm. Scale bars, 100 µm.

File Name: Supplementary Movie 7
Description: Time-lapse movie of the droplet containing 200 µM cytochalasin D (Supplementary Figure 5a). The cluster was not assembled, and the wave did not occur, indicating that actin polymerization is essential for cluster formation and actomyosin wave generation. Actin polymerization was initiated at 0 s. The height of the chamber was 100 µm. Scale bars, 100 µm.

File Name: Supplementary Movie 8
Description: Time-lapse movie of the droplet containing 10 µM phalloidin (Supplementary Figure 5b). The cluster was formed and actomyosin waves were generated several times, followed by cessation of wave generation, indicating that actin depolymerization is essential to maintain periodic actomyosin waves. Actin polymerization was initiated at 0 s. The height of the chamber was 100 µm. Scale bars, 100 µm.

File Name: Supplementary Movie 9
Description: Time-lapse movie of the side view of the droplet. The images were reconstructed from Z-stack confocal images. Scale bars, 50 µm.

File Name: Supplementary Movie 10
Description: Time-lapse movie of cluster movement from the droplet boundary to the center. The cluster started to move at 0 s. The height of the chamber was 60 µm. Scale bars, 100 µm.

File Name: Supplementary Movie 11
Description: Time-lapse movie of cluster movement from the droplet center to the edge. The cluster was started to move at 0 s. The height of the chamber was 60 µm. Scale bars, 50 µm.

File Name: Supplementary Movie 12
Description: Time-lapse movie of the cluster oscillation. The coexistence of actomyosin waves and radial actomyosin bridges indicates that cluster oscillation is driven by these two distinct actomyosin dynamics. The movie was recorded approximately 5 min after the cluster formation. The height of the chamber was 60 µm. Scale bars, 100 µm.

File Name: Supplementary Movie 13
Description: Time-lapse movie of the symmetry breaking of cluster positioning observed by a confocal microscope. The radial actin bridges appear to be connected between the cluster and the droplet boundary. Rapid movement of the cluster toward the droplet boundary indicates that the off-centered cluster positioning is driven by the contraction of radial actin bridges. Movement was initiated from ~1 min after encapsulation. The height of the chamber was 30 µm. Scale bar, 50 µm.

File Name: Supplementary Movie 14
Description: Time-lapse movie of the motion of the cluster after cutting the actomyosin bridges observed by a confocal microscope. The height of the chamber was 30 µm. Scale bar, 50 µm.

File Name: Supplementary Movie 15
Description: Time-lapse movie of the droplet containing 250 nM α-actinin. The cluster was assembled at 0 s. The height of the chamber was 100 µm. Scale bars, 100 µm.

File Name: Supplementary Movie 16
Description: Time-lapse movie of the droplet containing 300 nM gelsolin. The cluster was assembled at 0 s. The height of the chamber was 100 µm. Scale bars, 100 µm.

File Name: Supplementary Movie 17
Description: Time-lapse movie of the droplet containing 30 nM mDia2. The cluster was assembled at 0 s. The height of the chamber was 100 µm. Scale bars, 100 µm.

File Name: Supplementary Movie 18
Description: Numerical simulation of cluster positioning dynamics, with parameters: $D = 120$ µm, $L = 8$ µm, $\tau = 1$ s. The yellow cross mark represents the bulk actin network being crosslinked. Peripheral contraction of the cluster was initiated, when all of the crosslinking-sites in bulk space $N = R/L = D/(2L)$ were occupied by crosslinkers. The cluster was initially in the center and was finally entrapped at the edge.

File Name: Supplementary Movie 19
Description: Numerical simulation of cluster positioning dynamics, with the following parameters: D = 300 μm, L = 8 μm, τ = 1 s. The yellow cross symbol represents the bulk actin network being crosslinked. The cluster is initially in the edge, and finally entrapped at the center.

File Name: Supplementary Movie 20
Description: Actomyosin waves in non-circular geometry chambers. The height of the chamber was 60 μm. Scale bars, 100 μm.

File Name: Supplementary Movie 21
Description: Time-lapse movie of the migrating droplet containing 10 nM His-VCA emulsified with 10% DGS-NTA(Ni) in eggPC. The height of the chamber was 100 μm. Scale bar, 100 μm.
